# Anti-Angiogenic Treatment in Pseudomyxoma Peritonei—Still a Strong Preclinical Rationale

**DOI:** 10.3390/cancers13112819

**Published:** 2021-06-05

**Authors:** Yvonne Andersson, Karianne G. Fleten, Torveig W. Abrahamsen, Wenche Reed, Ben Davidson, Kjersti Flatmark

**Affiliations:** 1 Department of Tumor Biology, Norwegian Radium Hospital, Oslo University Hospital, P.O. Box 4953 Nydalen, 0424 Oslo, Norway; Yvonne.Andersson@sykehusapotekene.no (Y.A.); kaflet@rr-research.no (K.G.F.); Torveig.Weum.Abrahamsen@rr-research.no (T.W.A.); 2 Department of Pathology, Norwegian Radium Hospital, Oslo University Hospital, P.O. Box 4953 Nydalen, 0424 Oslo, Norway; WRE@ous-hf.no (W.R.); ben.davidson@ous-hf.no (B.D.); 3 Department of Research, Innovation and Education, Norwegian Radium Hospital, Oslo University Hospital, P.O. Box 4953 Nydalen, 0424 Oslo, Norway; 4 Institute of Clinical Medicine, University of Oslo, 0310 Oslo, Norway; 5 Department of Gastroenterological Surgery, Norwegian Radium Hospital, Oslo University Hospital, P.O. Box 4953 Nydalen, 0424 Oslo, Norway

**Keywords:** pseudomyxoma peritonei, angiogenesis, in vivo, peritoneal metastases, peritoneal carcinomatosis

## Abstract

**Simple Summary:**

Patients with pseudomyxoma peritonei that are not cured by the standard treatment (cytoreductive surgery and hyperthermic intraperitoneal chemotherapy) have no efficacious treatment options. Drugs that inhibit formation of new vessels (anti-angiogenic drugs) could be a therapeutic option for these patients. Using patient samples and animal models we show that angiogenesis is important in pseudomyxoma peritonei and that anti-angiogenic drugs may indeed have an effect. Our results support continued efforts to determine the role of anti-angiogenic treatment in pseudomyxoma peritonei.

**Abstract:**

Pseudomyxoma peritonei (PMP) is a rare, slow-growing cancer characterized by progressive accumulation of intraperitoneal mucinous tumor deposits. Cytoreductive surgery and hyperthermic intraperitoneal chemotherapy (HIPEC) cures approximately 50% of patients, but in unresectable and recurrent cases, treatment options are limited. Anti-angiogenic treatment is being explored as a potential therapeutic option. Using PMP patient samples, microvessel densities (immunostaining for CD31 and CD105) and pro-angiogenic factors were analyzed, and the proliferative response upon incubation with human umbilical cord vascular endothelial cells (HUVEC) was determined. Growth inhibition by anti-angiogenic drugs was analyzed in patient-derived xenograft models of PMP. PMP tumor tissues were found to be highly vascularized and contained key pro-angiogenic factors, in particular related to vascular endothelial growth factor (VEGF) signaling, but interestingly, high levels of fibroblast growth factor 2 were also detected. HUVEC proliferation was stimulated upon incubation with fresh tumor samples and the observed proliferation could be inhibited by VEGF pathway inhibitor bevacizumab. In xenograft models the two VEGF pathway inhibitors, bevacizumab and aflibercept, inhibited tumor growth. This work reemphasizes the importance of angiogenesis as a major driver in PMP and strengthens the preclinical rationale for continued exploration of angiogenesis inhibition in the hope of providing novel treatment to a group of patients that have few other treatment options.

## 1. Introduction

Pseudomyxoma peritonei (PMP) is a rare, slow-growing abdominal cancer characterized by progressive accumulation of mucinous tumor tissue in the peritoneal cavity [1]. Curatively aimed treatment is offered at specialist centers and involves cytoreductive surgery (CRS) to remove all visible tumor tissue, followed by hyperthermic intraperitoneal chemotherapy (HIPEC) to kill microscopic residual disease [2,3]. However, almost half of PMP cases cannot be cured by CRS-HIPEC, and these patients have no efficacious treatment options. In this setting PMP is a debilitating and ultimately fatal disease, and patients with recurrent PMP experience a progressively decreasing quality of life caused by increasing burden of intraperitoneal tumor, leading to abdominal compression. There is a clear unmet need for treatment options to improve the cure rate of primary treatment and for palliation of recurrent disease.

Responses to systemic chemotherapy are generally disappointing in PMP, and biological agents, particularly angiogenesis inhibitors, have been suggested as an alternative therapeutic approach [4,5]. The clinical experience with anti-angiogenic treatment in PMP is, however, limited and findings are inconclusive. In a small cohort of recurrent PMP patients, a combination of capecitabine and vascular endothelial growth factor A (VEGFA) inhibitor bevacizumab (BEV) was well tolerated with median progression-free survival of 8.2 months [6]. No randomized trials have been conducted, but a large retrospective study of 130 non-resectable PMP cases that received either BEV in combination with chemotherapy or chemotherapy alone suggested a benefit of including BEV [7]. However, questions were later raised regarding differences in the chemotherapy regimens that may have confounded the interpretation, and therefore, the clinical utility of including BEV in this setting remains undetermined [8]. Importantly, no other anti-angiogenic treatment strategies have been studied in PMP, and there are no biomarkers available to suggest which drugs would be active. Additionally, although measurements of systemic levels of pro-angiogenic factors have been performed in animal models and humans, levels of these substances in PMP tissues have not been assessed.

We previously described the presence of vessels throughout the tumor tissue in surgical specimens from PMP patients and in patient-derived xenograft (PDX) models that were subsequently established from these specimens [9]. In this study, we further investigated angiogenesis and the presence of pro-angiogenic factors in tumor tissue from PMP patients and PDX models, analyzing the ability of tumor tissue to stimulate human umbilical cord vascular endothelial cell (HUVEC) proliferation. In addition, the efficacy of two anti-angiogenic drugs was examined in PDX models.

## 2. Results

### 2.1. Immunohistochemistry

**Figure 1:**
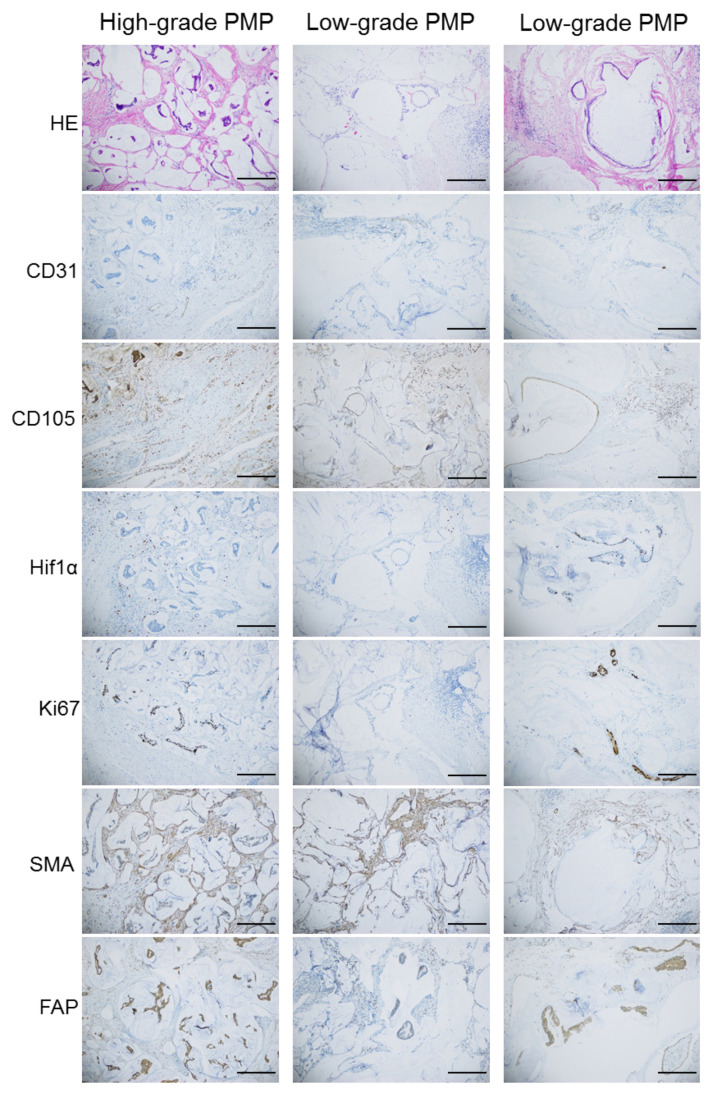
Representative HE and immunohistochemical images showing CD31, CD105, Hif1α, Ki67, SMA and FAP staining in samples from one patient with high-grade PMP and two with low-grade PMP. Scale bar indicates 300 µM.

The median score for Ki67 staining was 4 (min–max 3–5) in the high-grade (HG) and 2 (min–max 1–4) in the low-grade (LG) group; for Hif1α, the median score was 2.5 (min–max 1–4) for HG and 2 (min–max 1–5) for LG (Table 1 and Figure 1). The measurement of microvessel density (MVD) is a widely accepted method for assessing angiogenic activity through counting positive staining by anti-CD31 and anti-CD105 [10]. The median MVD assessed by quantifying presence of CD31 staining was 15.5 (min–max 9–28) in the HG and 19.5 (min–max 6–27) in the LG group. For CD105 positive vessels, the median count was 12 (min–max 6–21) for HG (*n* = 10) and 10 (min–max 4–19) for LG (*n* = 10). The HG group had a higher Ki67 score than the LG group (*p* < 0.05), otherwise no significant differences were observed. Smooth muscle actin (SMA) staining was observed throughout the stroma surrounding the tumor, and any variation between samples was explained by different amount of connective tissue. Fibroblast activating protein (FAP) staining was observed as diffuse staining of most of the tumor cells in all samples analyzed. SMA and FAP staining were therefore not quantified. Normal appendix stained with the same antibodies as control is shown in Figure S1.

### 2.2. Measurement of Pro-Angiogenic Factors

All the analyzed patient tumor samples (*n* = 14) contained pro-angiogenic factors (Table 2a). Normal human serum levels are not a validated reference for peritoneal samples, but were used for comparison because no reports of measurements in similar normal tissues were available. Interestingly, for VEGFA, placental growth factor (PlGF), fibroblast growth factor 2 (FGF2) and soluble Fms-like Tyrosine 1 (sflt1), the measured values were 8–780 times higher than reported serum values. For VEGFC and VEGFD, measured values were marginally higher (1.5–1.7×), while for angiopoietin1 and tyrosine-protein kinase receptor (Tie2), levels were lower than otherwise detected in normal serum.

In the PDX models, human and murine VEGFA and PlGF were measured in tumor tissue (Table 2b). High levels of hVEGFA (15,000–34,000 pg/mL,) but no hPlGF were detected. The mVEGFA levels varied between 700–1240 pg/mL, and mPlGF levels were 1100–2800 pg/mL. In serum samples from tumor bearing animals, hVEGFA was 0–500 pg/mL, while h/mPlGF and mVEGFA were not detectable, and in animals without tumor, neither factor was detectable in serum.

### 2.3. Incubation with PMP Tumor Samples Increased HUVEC Proliferation Which Could Be Inhibited by Anti-Angiogenic Drugs

**Figure 2:**
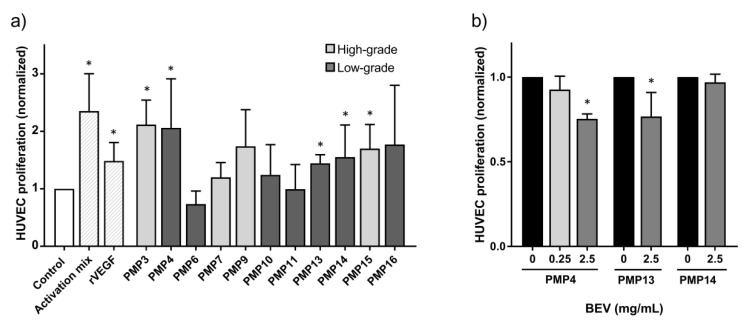
Proliferation of human umbilical cord vascular endothelial cells (HUVEC). Error bars indicate standard deviation. *, *p* ≤ 0.05. (**a**) proliferation measured after incubation with activation mix, recombinant VEGFA (rVEGF, 250 ng/mL) or tumor samples from patients with pseudomyxoma peritonei (PMP) compared to control medium. Light grey bars indicate high-grade, while dark grey indicate low-grade patient samples. (**b**) the proliferative response of HUVEC incubated with the PMP4, PMP13 and PMP14 tumor sample with and without addition of anti-angiogenic drug bevacizumab (BEV, 0.25 or 2.5 mg/mL).

Baseline proliferation upon incubation with control medium was used for comparisons, and increased proliferation was observed upon incubation with positive controls recombinant (r) VEGFA (1.5× control) and activation mix (2.5× control medium). For most of the 11 fresh patient tissue samples, increased, but variable HUVEC proliferation was observed. For five patient samples the increase was statistically significant compared to baseline (*p* < 0.05; Figure 2a), with two of the samples achieving close to maximum increase of proliferation (>2× control medium). In a subset of three samples, we further examined if the increased proliferation could be inhibited by the anti-angiogenic drug BEV (Figure 2b). A significant reduction was observed after treatment with 2.5 mg/mL BEV with the PMP4 and PMP13 samples.

### 2.4. In Vivo Activity of Anti-Angiogenic Drugs

**Figure 3:**
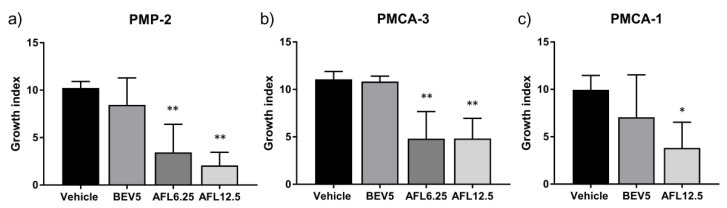
Responses to anti-angiogenic drugs bevacizumab (BEV, mg/kg) and aflibercept (AFL, mg/kg) treatment in patient derived xenograft models of pseudomyxoma peritonei, (**a**) PMP-2, (**b**) PMCA-3 and (**c**) PMCA-1. Error bars indicate standard deviation. *, *p* < 0.01; **, *p* < 0.001 for comparisons with vehicle treatment.

Tumor take was 100% in vehicle-treated animals in all models, PMP-2, PMCA-1 and PMCA-3. With BEV 5 mg/kg, variable non-significant inhibition of tumor growth was observed in all models (Figure 3a–c). In contrast, AFL treatment resulted in strong inhibition of tumor growth (*p* < 0.01) (Figure 3a–c).

## 3. Discussion

A dominating feature of PMP is the extraordinary abundant production and secretion of mucin to the extracellular space by a relatively low number of epithelial tumor cells. In fact, the bulky mucin is generally the cause of morbidity and mortality in PMP, rather than the volume of tumor tissue. One might think that with a low number of tumor cells, adequate nourishment and oxygen could be supplied by diffusion in a tissue of this type, but our findings illustrate that these are highly vascularized tissues. Vessels were positive for the pan-endothelial marker CD31, but also for CD105, which is recognized as a proliferation-associated endothelial marker signifying active angiogenesis, known to be induced by hypoxia [11]. Results from a recent study of 50 colorectal cancer cases (mean MVD of 24.2 and 19.2 for CD105 and CD31, respectively) showed only slightly higher MVD levels compared to our findings in PMP (median MVD of 11 and 18 for CD105 and CD31, respectively) [12]. Hypoxia induced transcription factor Hif1α was detected in most cases, which is consistent with hypoxia being a major driver of the angiogenic process in PMP, and Hif1α is a known up-regulator of several pro-angiogenic factors, including VEGFA, VEGFB, VEGFC, PlGF, FGF2 and flt1 [13,14]. The difference in Ki67 score indicated increased proliferation in HG compared to LG cases, which is to be expected, but this difference was not accompanied by a detectable difference in hypoxia or MVD. The results show the presence of angiogenesis in PMP tumor tissue at a level which is comparable to that observed in solid tumors, and suggest that the process could be partly driven by hypoxia.

A broad range of pro-angiogenic factors were detected in the PMP tumor samples; high levels were observed for some factors (VEGFA, PlGF, FGF2 and sflt1), moderately increased (VEGFC and VEGFD) or low levels (angiopoietin1 and Tie2) for others, compared to values measured in serum samples from healthy humans (Table 2a). Particularly high levels were detected for VEGFA, with a median concentration of 1660 pg/mL. In comparison, in a large study of patients with ascites caused by different pathological conditions (*n* = 1012), mean VEGFA values of 676 pg/mL and 218 pg/mL were reported in malignant and benign cases, respectively [15]. In a smaller study of gastric cancer patients (*n* = 35), the median VEGFA value was 660 pg/mL in ascites [16]. In our tumor samples, the median PlGF level was 469 pg/mL. Elevation of PlGF was previously detected in the peritoneal fluid samples from patients with endometriosis compared to ovarian cystadenomas, median 189 pg/mL and 88 pg/mL, respectively (*p* < 0.001) [17]. A novel finding was that in addition to the VEGF/VEGFR pathway members, high levels of FGF2 (median 1558 pg/mL) were detected. This potent pro-angiogenic growth factor interacts with FGFR expressed on endothelial cells and functions in synergy with VEGF signaling to promote angiogenesis. Elevated FGF2 serum levels have been reported in cancer patients compared to healthy individuals, but not exceeding 25 pg/mL, suggesting that the detected levels in PMP tissues are quite high [18]. Upon recognizing resistance development to inhibition of VEGF signaling alone, several drugs have been approved for other indications (such as nitendanib, pazopanib and regorafenib) that simultaneously target FGFR signaling in addition to the VEGF pathway [18,19]. We previously showed that regorafenib inhibited tumor growth in PDX models of PMP, suggesting that these drugs could be relevant in PMP [20]. Taken together, acknowledging the potential differences in sample processing and assay conditions, the tumor tissue analyses point to several opportunities for angiogenesis inhibition in PMP.

Interestingly, in tumor samples from the PDX models, hVEGFA was detected at high levels compared to the murine version, suggesting that a substantial part of the pro-angiogenic stimulus from VEGFA originated in the tumor cells. In contrast, only the murine version of PlGF was detectable in tumor tissue, which would indicate that any contribution of PlGF to the angiogenic process could be attributed to the stroma, rather than the tumor cells. Our findings are consistent with results from studies of PDX models in other cancers where tumor cells were shown to be the major source of VEGFA while PlGF was primarily produced by tumor stroma [21]. In serum, hVEGFA was detected while levels of mVEGFA were negligible, and neither human nor murine PlGF were detectable. In a previous study that included two PDX-models, serum mVEGFA and hPlGF were elevated compared to levels in non-tumor bearing animals, while serum hVEGFA and mPlGF and tissue levels were not measured [4]. Tissue levels of these factors could in principle be influenced by local and systemic interaction between pro-angiogenic factors, as VEGFA is known to enhance the expression of other pro-angiogenic factors, such as PlGF, and PlGF in turn has been shown to up-regulate VEGFA [22]. The results suggest that PDX models may be used to understand which cells are the main contributors to angiogenic stimulation, and tissue analysis could point to which pro-angiogenic factors to target.

The proliferative response observed when HUVECs were incubated with PMP tissue samples is consistent with the presence of pro-angiogenic factors in the tumor tissue. Furthermore, the response could be inhibited by BEV, suggesting that its target, VEGFA, could in part explain the proliferative response. The ex vivo findings are supported by the in vivo results from three PDX models, where BEV and AFL to variable extent inhibited tumor growth compared to vehicle treatment. Neither drug is completely specific for one pro-angiogenic factor, although VEGFA is the main target of BEV and AFL is known to target VEGFA, VEGFB and PlGF. Based on the tissue measurements, VEGFA and PlGF should be considered highly relevant targets for angiogenic inhibition in PMP, and both drugs would be relevant in this context, although limitations to the interpretation of these results must be kept in mind when translating findings in preclinical models to human subjects. AFL demonstrated greater antitumor activity than BEV in the in vivo experiments, which unexpectedly did not give rise to significant differences compared to vehicle treatment, although there was a clear trend. Increased survival after treatment with BEV has previously been observed by other investigators in PMP models [4]. There was no clear correlation between the observed inhibition of tumor growth and measured levels of hVEGFA, mVEGFA and mPlGF in the respective tumor tissues, but tissues from all the PDX models had detectable levels of these pro-angiogenic factors.

The physical properties of PMP tissues were essential to the execution of these studies, but also represent a potential source of bias and point to some important limitations. Surgical PMP specimens are often composed of a mixture of solid tissue and liquefied mucin, in both cases composed mainly of mucin with varying number of epithelial tumor cells and containing a vascular network, as shown in this work. For these studies, liquefied mucin samples were chosen, which made pipetting of samples possible without chemical pretreatment to disaggregate the tissues. Some samples (such as PMP 4, Figure 2b) could be frozen and thawed while retaining the pro-angiogenic properties, while other samples had activity only when fresh. This greatly limited the possibility of repeating experiments with tissue from many of the patients. The consistency of the tissues allowed analysis using standardized ELISA kits, which has not been undertaken previously on PMP tissues, but interpretation of results was challenging, since no directly comparable studies were available.

## 4. Materials and Methods

### 4.1. Patient Samples

Tumor samples were collected from patients undergoing CRS-HIPEC at the Norwegian Radium Hospital, part of Oslo University Hospital Comprehensive Cancer Center as part of the translational research program for peritoneal surface malignancies. For immunohistochemical analyses, 10 HG and 10 LG PMP cases (classified according to the Peritoneal Surface Oncology Group International (PSOGI) recommendation) [23] were selected from the institutional biobank. Fresh tumor samples (mucinous ascites) were collected from 14 patients, 4 HG and 10 LG cases. The study was approved by the regional ethics committee of South-East Norway (study approval #2010/2390) and written informed consent was required for participation. Resected tissue was processed for routine histopathological assessment, and histological examination of all tissue specimens was performed by an experienced pathologist.

### 4.2. PDX Models

All procedures and experiments involving animals were approved by the Norwegian Food Safety Authority (application ID # 4729), and were conducted according to the recommendations of the European Laboratory Animals Science Association. Female athymic foxn 1^nu^ mice, 6–8 weeks old, bred at Department of Comparative Medicine, Oslo University Hospital were used for the experiments. The establishment of PDX models PMP-2, PMCA-1 and PMCA-3 was previously described [9,24]. Briefly, the models were established from tumor tissue samples collected at the time of surgery for PMP. Tumor tissue was implanted intraperitoneally (i.p) in mice through a small midline incision. The PMP-2 and PMCA-3 were derived from patients with appendiceal primaries, while PMCA-1 was established from a patient with a primary mucinous rectal carcinoma. Mucinous tumor tissue is passaged to new generations of mice by simple injection. Additional details regarding the models have been summarized in Supplementary Table S1.

### 4.3. Storage and Handling of Fresh Patient and Xenograft Samples

Tumor samples from patients and PDX models were aliquoted and conserved at −80 °C until analysis. Thawed samples were diluted (1:10 and 1:20) in MCDB 131 cell medium (Thermo Fisher Scientific, Waltham, MA, USA) without additions for ELISA and with 1% fetal calf serum (FCS), 100 μg/mL penicillin and 100 μg/mL streptomycin, designated “control medium” for HUVEC experiments, and the samples were passed through a syringe (18 G and 21 G) 10 times to reduce viscosity just before experiments.

### 4.4. Immunohistochemical Analysis and Quantification of Microvessel Density

Formalin-fixed paraffin-embedded sections from the main surgical specimens were used for immunohistochemical analysis. Immunostaining was performed using antibodies targeting CD31 (panendothelial marker) (1:40, #M0823, Dako, Glostrup, Denmark), CD105 (proliferating endothelium marker) (1:25, NCL-CD105, Novocastra/Leica, Newcastle upon Tyne, UK), HIF1α (hypoxia marker) (1:300, #H72320-150, BD Bioscience, San Jose, CA, USA), Ki67 (proliferation marker) (1:25, #M7240, Dako), FAP (protease expressed on activated myofibroblasts and some cancers) (1:100, #ab53066, Abcam) and SMA (smooth muscle and myofibroblast marker)(1:1500, #M0851, Dako). Visualization was achieved using the EnVision+ peroxidase system (Dako), as recently described [24]. Negative controls consisted of sections that underwent similar staining procedures with non-relevant rabbit immunoglobulins or a monoclonal antibody of the same isotype as the primary antibody used. For all antibodies, positive controls were included with satisfactory results. The immunoreactivity of Ki67 and HIF1α was quantified by scoring the staining from 0 to 5 according to the distribution of positive cells (0 = 0%, 1 = <1%, 2 = 1–10%, 3 = 11–33%, 4 = 34–66% and 5 = 67–100%). Immunostaining of CD105 and CD31 was used to determine MVD, by the so-called “hot-spot” method [25]. Vessel count was assessed by light microscopy in tumor areas containing the highest numbers of capillaries and was performed independently by two pathologists (WR and BD). Vessel count was performed at 400× magnification field and the average of three representative areas was determined as the MVD; minor disagreements with respect to scoring were solved by re-examination and establishment of consensus. For CD31 a vessel lumen was necessary for a structure to be defined as a vessel, whereas for CD105 this was not required.

### 4.5. Measurement of Pro-angiogenic Factors in Tumor Samples from Patients and PDX Models

Concentrations of pro-angiogenic factors in patient and PDX samples were quantified using two ELISA platforms. The electrochemiluminescence detection with Meso Scale Discovery (MSD) V-PLEX Angiogenesis Panel 1 Human Kit (VEGF-A/-C/-D, PlGF, bFGF/FGF2, sflt1/VEGFR1, Tie2) was performed according to the manufacturer’s protocol (MSD, Hercules, CA, USA). In addition, human pro-angiogenic factors VEGFA, PlGF and angiopoietin1 and mouse VEGFA and PlGF were analyzed with specific ELISA plates according to the manufacturer’s protocol (R&D Systems, Inc, Minneapolis, MN, USA). After thawing, samples were handled as described above and centrifuged briefly at 2000× *g* for 2 min at 4 °C. The supernatants were incubated on MSD V-plex or the specific ELISA plates. MSD V-plex plates were washed and read using SECTOR Imager 2400 software (MSD) and the ELISA (R&D Systems, Inc) on a plate reader Victor 1420 Multilabel Counter, (Wallac/PerkinElmer Life Sciences, Turku, Finland) at 450 nm with wavelength correction at 570 nm.

### 4.6. HUVEC Culture Conditions

Primary cultures of HUVEC (kindly provided by Dr. Guttorm Haraldsen, Oslo University Hospital, Norway) were considered as passage 1 and aliquots were stored at −80 °C. HUVEC were grown in a supplemented MCDB 131 cell medium, designated “activation mix” (containing glutamax, hepes (all from Lonza, Basel, Austria), heat-inactivated FCS (7.5%, PAA, GE Healthcare, UK)), FGF2 (1 ng/mL, PeproTech Nordic, Stockholm, Sweden), human *epidermal growth factor* (EGF) (10 ng/mL, R&D Systems, Inc.), hydrocortisone (1 µg/mL, Sigma, St. Louis, MO, USA), 100 μg/mL penicillin and 100 μg/mL streptomycin) at 37 °C, 5% CO_2_ humidified incubator. For the proliferation assay, the control medium was supplemented with drugs or tumor samples according to the experimental condition tested. Confluent cell monolayers were exposed to trypsin (0.25%) for 2 min to detach the cells, whereupon the trypsin was neutralized with control medium. Detached cells were centrifuged at 1000 rpm for 5 min before used in experiments, or frozen down. HUVEC were used up to passage 6.

### 4.7. HUVEC Proliferation and Angiogenic Inhibition Assay

HUVECs were thawed and resuspended in activation mix, and seeded in 96-well tissue culture plates (1 × 10^4^ cells/well). After 6 h, fresh control medium was added to “starve” the cells for 18–20 h. The day after, the medium was replaced with fresh control medium, activation mix (positive control), or control medium supplemented with recombinant (r) human VEGFA (250 ng/mL, PeproTech Nordic), or diluted mucinous tumor tissue from PMP patients and then incubated for 72 h. Determination of cell viability was performed using the MTS assay as previously described [26]. The HUVEC proliferation assay results were calculated from six replicates in each experiment with three to eleven independent experiments (Figure 2a). To assess the effect of adding anti-angiogenic drugs, tissue samples were pre-incubated with BEV (0.25 or 2.5 mg/mL) at 37 °C for 2 h before transfer to the HUVEC cells. HUVECs were then incubated, as described above. The experiment was repeated at least 4 times.

### 4.8. In Vivo Experiments

For treatment experiments, 250 µL mucinous tumor tissue from the PMP-2, PMCA-1 or PMCA-3 models was injected i.p, and i.p. injections of anti-angiogenic drugs, BEV (5 mg/kg) and AFL (6.25 and 12.5 mg/kg) or vehicle were initiated the following day to simulate the clinical situation after CRS with a very low remaining tumor load in the peritoneal cavity. Treatments were administered twice weekly. The drug doses used were selected based on available literature. Mice were randomly assigned to treatment groups of 6 mice. The mice were sacrificed when abdominal distention was observed, and typically, 4–5 g of mucinous tumor tissue was present when this occurred. Animals with no sign of tumor growth were sacrificed 100 days (min–max 99–106 days) after experiment initiation, which in all experiments was at least twice the median time of the survival of the vehicle treated animals. Tumor-bearing mice in these models develop abdominal distension and rarely symptoms of tumor growth. Since abdominal distension can be difficult to assess objectively, tumor growth/response was quantified by calculating a growth index as previously described [27], by combining the key parameters survival time (in days) and tumor load at the time of sacrifice (in g) to one parameter, using the equation:

Growth index = tumor weight + ((T_total_ − T_A_)/T_Total_) × 10

where T_A_ is the time from start of the experiment until sacrifice of the animal, and T_Total_ is the total duration of the experiment. One PMCA-1 control was excluded from the analysis due to no tumor take.

### 4.9. Statistical Analyses

Statistical analyses were conducted using GraphPad Prism v7 (GraphPad Software, LaJolla, CA, USA) and Student’s t-tests. *p*-values < 0.05 were considered statistically significant.

## 5. Conclusions

We have demonstrated that angiogenesis is an essential property of the PMP disease entity, independent of histological subgroup. Pro-angiogenic factors were detectable in tumor tissue samples from patients and PDX models, and ex vivo, PMP tissue samples induced HUVEC proliferation that could be inhibited by BEV. Finally, BEV and AFL were shown to inhibit tumor growth in PDX models of PMP. In future studies, it would be of interest to investigate drugs that target pathways other than the VEGF/VEGFR axis (for instance inhibition of the FGF signaling pathway), drugs other than antibodies (such as tyrosine kinase inhibitors), and also drug combinations that have demonstrated efficacy in other cancer entities [19]. Ex vivo and in vivo model systems may be useful to aid selection of the most promising inhibition strategies. Taken together, this work strengthens the rationale for continued clinical exploration of angiogenesis inhibition in PMP, hoping to provide novel treatment to patients that have few other therapeutic options.

## Figures and Tables

**Table 1:** Immunohistochemical analysis of tumor samples from pseudomyxoma peritonei (PMP) patients.

Markers	High-Grade PMP (*n* = 10)		Low-Grade PMP (*n* = 10)	
	Median	Min–Max	Median	Min–Max
CD31	15.5	9–28	19.5	6–27
CD105	12	6–21	10	4–19
Hif1α	2.5	1–4	2	1–5
Ki67	4 ^†^	3–5	2 ^†^	1–4

^†^ The high-grade group had a higher Ki67 score than the low-grade group (*p* < 0.05).

**Table 2:** Pro-angiogenic factors in tumor samples from pseudomyxoma peritonei (PMP) patients (2a) and the patient-derived xenograft (PDX) models (2b).

**(a)**	**^†^ Patient Tumor Tissue (pg/mL)**		**^#^ “Normal” Human Serum Values (pg/mL)**	
	**Median**	**Min–Max**	**Median**	
VEGFA	1660	0–29,225	194	
VEGFC	307	0–3107	208	
VEGFD	1618	93–2337	929	
PlGF	469	124–1898	6	
FGF2	1558	11–27,591	<2	
Angiopoietin	5707	900–27,591	37,122	
sflt1	2230	544–11,539	126	
Tie2	1258	0–2336	6153	
**(b)**	**^‡^ PDX Tumor Tissue (pg/mL)**			
	**Median**			**Min–Max**
hVEGFA	22,333			15,476–34,237
mVEGFA	780			700–1240
hPlGF	nd			nd
mPlGF	2447			1133–2848

^†^, *n* = 14; ^‡^, *n* = 3; ^#^ Normal human serum levels were derived from the respective manufacturer’s protocol. VEGF, vascular endothelial growth factor; PlGF, placental growth factor; FGF2, fibroblastic growth factor 2; Tie2, tyrosine-protein kinase receptor (angiopoietin receptor); Sflt1, soluble fms-like tyrosine kinase-1 (or sVEGFR-1); h, human; m, mouse; nd, not detected.

## Data Availability

Data is contained within this article and supplementary files.

## Supplementary Materials

The following are available online at https://www.mdpi.com/article/10.3390/cancers13112819/s1, Figure S1: Immunohistochemical images showing HE, CD31, CD105, Hif1α, Ki67, SMA and FAP staining in normal appendix as a control to staining’s shown in Figure 1. Scale bar indicate 300 µM. Table S1: Properties of the PDX models used in experiments: Patient characteristics; immunohistochemistry (IHC); mutation analysis by targeted next-generation sequencing.
