# Supplementary material for: Anti-Angiogenic Treatment in Pseudomyxoma Peritonei—Still a Strong Preclinical Rationale
Source: Cancers (Basel). 2021 Jun 5;13(11):2819. doi: 10.3390/cancers13112819 (PMC8201024; doi:10.3390/cancers13112819)
Supplement: Supplementary file 1 [file cancers-13-02819-s001.zip › cancers-1237088-supplementary.pdf]

cancers

MDPI

Supplementary Materials

Anti-Angiogenic Treatment in Pseudomyxoma Peritonei—Still a Strong Preclinical Rationale

Yvonne Andersson, Karianne G. Fleten, Torveig W. Abrahamsen, Wenche Reed, Ben Davidson and Kjersti Flatmark

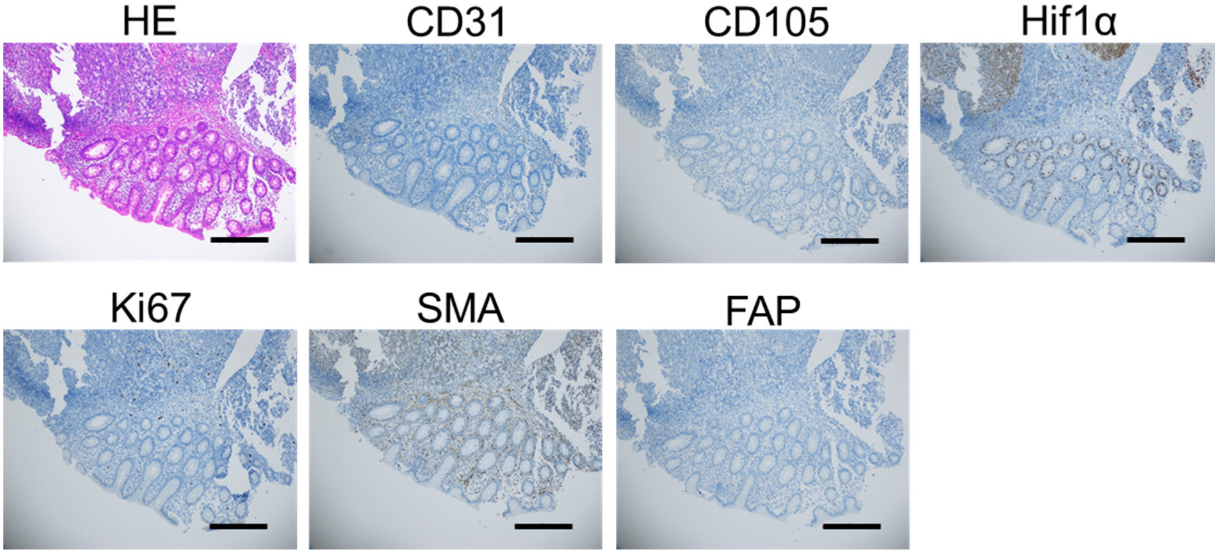

Figure S1. Immunohistochemical images showing HE, CD31, CD105, Hif1α, Ki67, SMA and FAP staining in normal appendix as a control to stainings shown in Figure 1. Scale bar indicate 300 µM.

Table S1. Properties of the PDX models used in experiments: Patient characteristics; immunohistochemistry (IHC); mutation analysis by targeted next-generation sequencing. The number of immunoreactive tumor cells was semi-quantitatively evaluated:.0 (no positive cells); 1 = 1% to 5%; 2 = 6% to 25%; 3 = 26% to 75%; and 4 = 75% to 100%. The data was previously published in [1–3].

**Patient Characteristics**	**PMP-2**	**PMCA-1**	**PMCA-3**
Age	56	68	62
Gender	F	F	M
**IHC parameters**			
CK20	4	0	4
CK7	4	4	1
CEA	4	4	4
Vimentin	0	0	0
Ki67	2	3	3
VEGF	0	0	0
**Mutations**			
KRAS	G12V	G12A	*WT*
GNAS	R201C	*WT*	R201C
BRAF	*WT*	*WT*	V600E

References

1. Flatmark, K.; Davidson, B.; Kristian, A.; Stavnes, H.T.; Førsund, M.; Reed, W. Exploring the peritoneal surface malignancy phenotype—a pilot immunohistochemical study of human pseudomyxoma peritonei and derived animal models. *Human Pathology* **2010**, 41, 1109–1119.

2. Flatmark, K.; Reed, W.; Halvorsen, T.; Sorensen, O.; Wiig, J.N.; Larsen, S.G.; Fodstad, O.; Giercksky, K.E. Pseudomyxoma peritonei--two novel orthotopic mouse models portray the PMCA-I histopathologic subtype. *BMC Cancer* **2007**, *7*, 116, doi:10.1186/1471-2407-7-116.
3. Fleten, K.G.; Lund-Andersen, C.; Waagene, S.; Abrahamsen, T.W.; Mørch, Y.; Boye, K.; Torgunrud, A.; Flatmark, K. Experimental Treatment of Mucinous Peritoneal Metastases Using Patient-Derived Xenograft Models. *Translational oncology* **2020**, *13*, 100793.
